# Understanding p53 tumour suppressor network

**DOI:** 10.1186/s13062-021-00298-3

**Published:** 2021-08-06

**Authors:** Emanuele Panatta, Carlotta Zampieri, Gerry Melino, Ivano Amelio

**Affiliations:** 1 Department of Experimental Medicine, TOR, University of Rome Tor Vergata, 00133 Rome, Italy; 2 School of Life Sciences, University of Nottingham, Nottingham, UK

**Keywords:** Tumour suppression, DNA damage, Stress response, Cell death

## Abstract

The mutation of TP53 gene affects half of all human cancers, resulting in impairment of the regulation of several cellular functions, including cell cycle progression and cell death in response to genotoxic stress. In the recent years additional p53-mediated tumour suppression mechanisms have been described, questioning the contribution of its canonical pathway for tumour suppression. These include regulation of alternative cell death modalities (i.e. ferroptosis), cell metabolism and the emerging role in RNA stability. Here we briefly summarize our knowledge on p53 “canonical DNA damage response” and discuss the most relevant recent findings describing potential mechanistic explanation of p53-mediated tumour suppression.

## Canonical tumour suppression signalling: apoptotic cell death

**Fig. 1:**
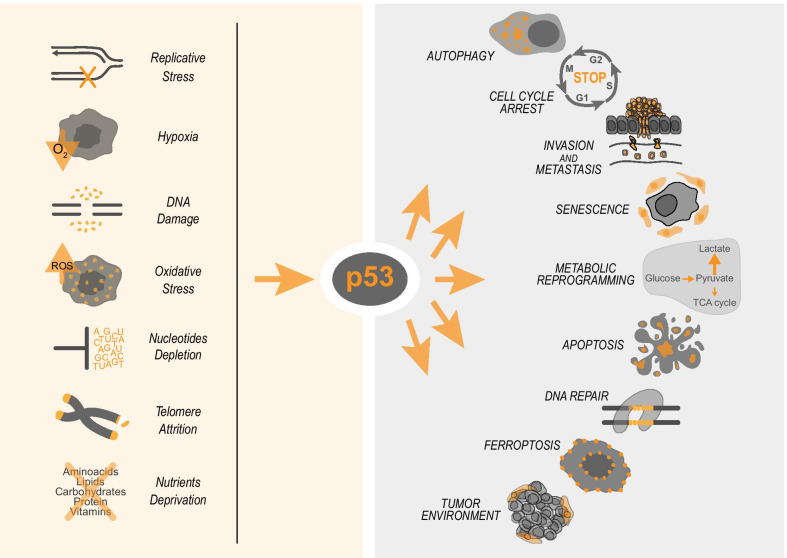
P53 drives the cellular adaptation to stress. Different types of cell stressors, such as DNA damage, nutrient deprivation, and replicative stress, can activate p53 which in turn drives specific responses depending on type and magnitude of stress. For example, in mild/moderate reversible DNA damage p53 can stop cell cycle progression and promote the DNA repair. When the DNA damage is severe p53 promotes apoptosis

Every second cancer carries an inactivating mutation in the TP53 gene. Nonetheless, despite 40 years of high-quality studies, the p53-regulated tumour suppressive programme is still a puzzle, and cell death/cell cycle arrest are still considered among the most relevant regulatory aspects (Fig. 1). Apoptosis is an ordered and tightly regulated form of cell death and is highly associated to prevention of tumorigenesis. Sequence and homology of p53 are evolutionarily conserved in *Drosophila melanogaster* (named *dmp53*) and *Caenorhabditis elegans* (named *cep-1*) [1–3], although debate is still open on whether the mammalian p53 family member, p63 better resembles biochemically and functionally the ancestor form of the protein [4–10]. The response to DNA damage, responsible of activating p53-mediated cell cycle arrest and apoptosis, is a dominant mechanism of p53-mediated network and strongly conserved across species, including *Drosophila melanogaster* and *Caenorhabditis elegans.* Experimental evidence however emerged over the past decade, questioning the simplistic interpretation that p53 tumour suppression mainly rely on the regulation of cell cycle arrest and apoptosis. Mouse models carrying DNA-damage insensitive p53 forms have shown that p53 can suppress tumour development in absence of cell cycle arrest and/or apoptosis induction [11–15]. In further support of this, p53 can prevent tumour development in the mouse lacking p21, Puma and Noxa, the major p53 downstream targets responsible for cell cycle arrest and apoptosis in DNA damaged cells [16]. Thus, these data have questioned the specific contribution of p53-mediated DNA damage response (here referred as “canonical” p53 tumour suppressive signalling), indicating that p53 network might be more complex and unexplored that previously anticipated [17].

The “non-canonical” p53 tumour suppressive signalling includes several biological processes that in stressed/damaged cells can influence tumour initiation or progression; these includes metabolic stress, epigenetic reprogramming, regulation of reactive oxygen species (ROS) response, autophagy [18]. Multiple stress signalling can indeed control p53 activation. Conditions such as nutrient deprivation, hypoxia, nucleotide depletion can lead to different degree of p53 activation, impinging on the biology of the cell at different level, with different mechanisms (Fig. 1) [19–23].

Although there is consensus that p53 acts by cooperating with a network of executors of its tumour suppression function, the spectrum and relative importance of individual players and the nature of their overlap and complementarity are unknown. Even less clear is the mechanism of the p53 mutant and its gain-of-function effects [24–28] and how interaction with extrinsic factors, such as microenvironment, microbiota and others, influences its function [29–32]. Thus, the mechanisms underlining the p53-dependent maintenance of genome integrity and tumour suppression remain fundamentally only partially known.

## p53 in ferroptotic cell death

Ferroptosis is associated to an iron-dependent peroxidation of membrane lipids [33] and can be triggered by GSH depletion or by direct inactivation of GPX4 [34–37], both involved in reduction of peroxidic species of polyunsaturated fatty acids. p53 has been implicated in both activation/inhibition of this cell death modality. Despite p53^3KR^ (K117R/K161R/K162R) mutant is transcriptionally inactive on p53 pro-apoptotic target genes, mice carrying p53^3KR^ do not develop tumours [38]. This phenotype was associated to the ability of p53^3KR^ to repress the cystine/glutamate antiporter xCT (SLC7A11), responsible for GSH synthesis, promoting ferroptosis. Hence, p53 tumour suppressive function was postulated to rely on the regulation of ferroptosis.

The calcium-independent phospholipase iPLA2β was also recently shown to critically regulate p53-dependent ferroptosis upon reactive oxygen species (ROS)-induced stress, a classic regulator of cellular functions [39–41]. Peroxidized lipids detoxification mediated by iPLA2β is sufficient to suppress p53-driven ferroptosis, while iPLA2β inhibition sensitizes tumour cells to p53-driven ferroptosis, promoting p53-dependent tumour suppression in xenograft mouse models [42].

p53 is also implicated in the metabolism of polyamines. The SAT1 gene is transcriptionally controlled by p53 [43]. SAT1 is an enzyme that regulates the rate of conversion of spermidine and spermine to putrescine. The transcriptional induction of SAT1 correlates with the expression of arachidonate 15-lipoxygenase (ALOX15), that participates in the peroxidation process. A recent study identified a p53-dependent ferroptosis pathway mediated by another member of the lipoxygenase family, ALOX12, an enzyme involved in lipid peroxidation. Following redox stress, p53 downregulates SLC7A11 and indirectly activates the ALOX12, increasing the intracellular levels of lipid peroxides [44]. Also, the p53 family transcriptional target glutaminase-2 (GLS-2) was associated to activation of ferroptosis. GLS2 converts glutamine into glutamate in a process called glutaminolysis [45–48]. GLS2 inhibition affects the ferroptosis process in fibroblasts [49]. How and whether GLS2 regulation of ferroptosis correlates with p53 tumour suppression is not clearly defined.

p53 was also implicated in the opposite role of repressing ferroptosis. A first example is the inhibition of the (dipeptidyl peptidase-4) DPP4 activity. DPP4 is an enzyme expressed on the cell surface where it can activate lipid peroxidation interacting with NADPH oxidase 1 (NOX1). In human colorectal cancer, p53 was shown to form a complex with DPP4, translocating it into the nucleus. Loss of p53 prevents DPP4 accumulation in the nucleus and facilitates plasma-membrane-associated DPP4-dependent lipid peroxidation, which finally results in ferroptosis [50].

## p53 metabolic regulation and lipids biosynthesis

The function of p53 in tumour suppression has emerged as a complex integration of multi biological processes [51]. Within these the regulation of cellular metabolism and autophagy in addition to influencing the ferroptosis process strongly impacts the ability of cell to respond and adapt to perturbation, preventing accumulation of damage that can lead to cancer.

p53 supports mitochondrial respiration by limiting glycolysis. p53 represses expression of the glucose transporters GLUT1, GLU3 and GLUT4 [52–54]. Moreover, p53 reduces expression of TIGAR, a regulator of glucose breakdown [55], and of PDK2 (pyruvate dehydrogenase kinase 2), which inactivates the pyruvate dehydrogenase complex [56], regulating access of pyruvate into the Krebs cycle.

p53 plays also in the metabolism of fatty acids and cholesterol. p53 promotes the oxidation of fatty acids, making them no longer available to cancer cells. In fact, it induces the expression of Lipin-1 which activates other genes involved in the oxidation of fatty acids [57]. Moreover, p53 blocks maturation of SREBP2 (sterol regulatory element binding protein 2) the master transcriptional regulator of biosynthesis of cholesterol and nonsterol isoprenoids. By using a mouse model of liver cancer the groups of Carol Prives and Scott Lowe demonstrated that downregulating mevalonate pathway p53 suppresses tumorigenesis of premalignant hepatocytes [58].

## p53 control of mRNAs processing via Zmat3

More recent genomic techniques are helping in dissecting the functional p53 tumour suppressing network. Through a shRNA in vivo screen, 166 known p53 target genes were silenced [59]. This experiment demonstrated that extensive functional overlap of several p53-regulated processes delineates a defence against cancer development and in particular DNA repair plays an essential role. Depletion of the DNA repair gene Mlh1 could recapitulate lymphoma in wt mice and enforced expression of Mlh1 was able to delay the tumour phenotype of p53^−\−^ mice. From this approached also *Zmat3* was implicated in development of lymphoma/leukaemia, but only when also *Puma* and *Cdkn1a* were depleted. Knockdown of *Zmat3* in p53^−/−^ hematopoietic stem/progenitor cells did not accelerate lymphoma in mice, reinforcing the hypothesis that this gene acts downstream of p53 [59]. Also, lack of *Zmat3 *per se is not sufficient to cause lymphoma/leukaemia, confirming that its loss can only promote tumour development when p53-dependent apoptosis or cell cycle arrest (via Puma and p21 respectively) are impaired [59].

A second in vivo genetic screening using RNAi and CRISPR technologies against 87 p53 targets, confirmed the importance of *Zmat3* in p53-dependent cancer biology [60]. Induction of tumour in *Kras*^*G12D*^-driven mouse lung adenocarcinoma and hepatocellular carcinoma depleted for *Zmat3* leads to larger tumour and greater tumour burden than in control mice, only when p53 was expressed. No alterations of the tumour size were observed in the same cancer models with p53 depletion [60]. By analysing a cohort of breast cancer patients, it was observed that low level of *ZMAT3* correlates with a reduction in the survival, only in wild-type *TP53* tumours. Furthermore, mutations and deletions in *ZMAT3 locus* are mutually exclusive with mutations and deletions in *TP53* gene in uterine corpus endometrial carcinoma [60].

**Fig. 2:**
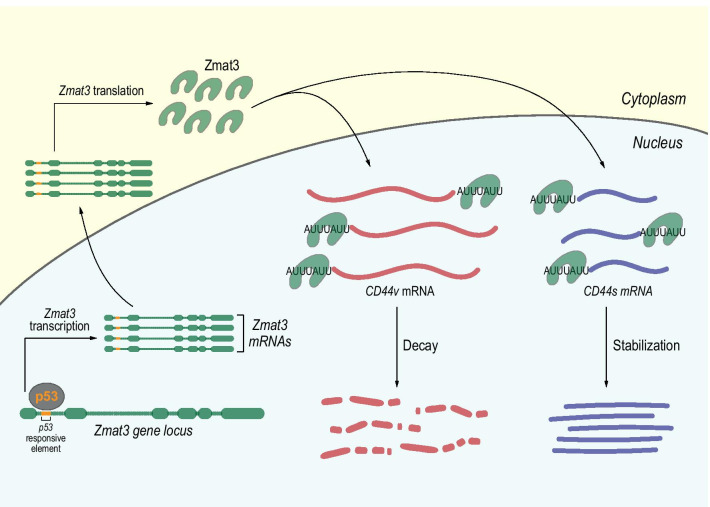
p53 in the control of mRNA stability. p53 can transcriptionally regulate Zmat3 by binding a p53-responsive element in the first intron of *Zmat3* gene. Zmat3 is localised in the nucleus and can bind AU-rich region of *CD44* mRNAs. The binding on the long and oncogenic CD44 isoform (*CD44v*) promotes its degradation by mRNA decay process. Conversely, by binding the shorter and non-oncogenic isoform (*CD44s*) Zmat3 promotes the mRNA stabilization. In this context, modulating the expression of Zmat3, p53 regulates the balance of CD44 isoforms

Mechanistically, Zmat3 *locus* contains a perfectly matched p53 responsive element (RE) in the first intron of both human and mouse gene, and it is directly bound by p53 in ChIP-seq data [61, 62] (Fig. 2). The disruption of the p53 RE by using specific sgRNAs in *E1A;Hras*^*G12V*^*;Cas9* MEFs, significantly reduces either p53 binding to Zmat3 *locus* and the levels of its RNA and protein [60]. ZMAT3 is a 32 KDa zinc-finger protein containing an RNA binding domain highly conserved across the evolution [63–65]. It localizes in the nucleus, where interacting with AU-rich 3′ untranslated regions, it can stabilize the mRNA targets [66] or promotes their decay [67]. One of the best characterized ZMAT3-target is *CD44*, potent oncogene and stem cell marker [68]. *ZMAT3* silencing led to the upregulation of the oncogenic and longer *CD44v* variant and downregulation of the standard, non-oncogenic, *CD44s* isoform (Fig. 2). Furthermore, *TP53* silencing recapitulates this phenotype, suggesting an important role for *TP53* in regulating the splicing of CD44 in cancer biology. These studies underline the importance of Zmat3 in the p53-dependent tumour biology, but above all, underline the complexity behind p53 function(s), which is still only partially understood.

## Conclusion

Despite over 40 years of studies dedicated to understanding the role of p53 in cancer, there are still key unanswered questions. These include for example the exact mechanisms by which p53 protects integrity of the somatic cell genome. Based on the dispensable role for tumour suppression of p53 mediated DNA damage response, the mechanism by which genomic instability is prevented remains elusive. The last decade has seen a massive expansion of cancer genomics studies, including development of predictive model of gene network [69–75] and improvement of experimental model of studies, including effective genetic editing techniques and 3D cell culture models [76–79]. With the support of the expansion of cancer genomics studies [70, 80–86] and methodological improvements [77, 87, 88], the determination of the p53 mediated gene network can lead not only to a greater understanding of tumour biology but also to design of more accurate anti-cancer therapies.

## Data Availability

Available upon requests.
